# Interventional Management of Intermediate-High-Risk Pulmonary Embolism: Current Evidence, Patient Selection and Personalised Treatment Strategies

**DOI:** 10.3390/jcm15135041

**Published:** 2026-06-28

**Authors:** Patrycja Paszenda, Agnieszka Kowalik, Izabella Profaska, Ewa Mroczek, Mateusz Garus, Robert Zymliński, Wiktor Kuliczkowski, Piotr Gajewski

**Affiliations:** 1 Faculty of Medicine, Wroclaw Medical University, 50-367 Wroclaw, Poland; 2 Student Scientific Organisation, Institute of Heart Diseases, Wroclaw Medical University, 50-367 Wroclaw, Poland; 3 Institute of Heart Diseases, Jan Mikulicz Radecki University Hospital in Wroclaw, Borowska 213, 50-556 Wroclaw, Poland

**Keywords:** pulmonary embolism, intermediate-high-risk, catheter-directed therapies, thrombus aspiration, PERT

## Abstract

Intermediate-high-risk (IHR) pulmonary embolism (PE) remains one of the most challenging clinical phenotypes in contemporary PE management. Although haemodynamically stable at presentation, these patients remain at significant risk of clinical deterioration, right ventricular failure, and haemodynamic decompensation. Current management primarily relies on anticoagulation and close surveillance, while routine reperfusion therapy remains controversial due to the balance between potential haemodynamic benefit and bleeding risk. Catheter-based reperfusion strategies, including catheter-directed thrombolysis and mechanical thrombectomy, have emerged as potential alternatives to systemic thrombolysis in selected patients. However, despite growing procedural adoption, important uncertainties remain regarding optimal patient selection, timing of intervention, and comparative effectiveness. Current evidence demonstrates consistent short-term improvements in surrogate haemodynamic parameters, but robust evidence for reductions in mortality, recurrent PE, chronic thromboembolic complications, or long-term functional impairment remains limited. This narrative review critically evaluates contemporary catheter-based reperfusion strategies in IHR PE, focusing on methodological limitations of the current evidence base and unresolved challenges in clinical decision-making. It highlights the limitations of existing risk stratification models and the heterogeneity of IHR PE, where static risk categories often fail to capture dynamic clinical deterioration. Future progress in this field will likely depend on improved patient selection, refined risk assessment, and personalised reperfusion strategies supported by high-quality comparative trials.

## 1. Introduction

Pulmonary embolism (PE) remains one of the leading causes of cardiovascular mortality globally and constitutes a significant clinical and public health challenge [1]. High-risk PE, characterised by overt haemodynamic instability, necessitates immediate reperfusion therapy, whereas low-risk PE is managed with pharmacological treatment. The optimal management of intermediate-high-risk (IHR) PE, however, remains uncertain [2,3].

IHR PE is characterised by right ventricular (RV) dysfunction and myocardial injury in the absence of systemic hypotension [3,4,5,6]. Importantly, IHR PE represents a population with highly variable risks of deterioration and treatment response [7,8,9,10]. Although patients are haemodynamically stable at presentation, they remain at considerable risk for early clinical deterioration, circulatory collapse, and mortality [6,11,12]. The current standard of care, anticoagulation, may not provide immediate reduction in thrombotic burden or RV afterload [2,13]. Systemic thrombolysis in intermediate-risk PE is constrained by a significant risk of major bleeding, including intracranial haemorrhage (ICH), and lacks a clear mortality benefit in haemodynamically stable patients [13,14,15]. Consequently, IHR PE represents a therapeutic grey zone, requiring clinicians to balance the risk of haemodynamic deterioration against the potential complications of reperfusion therapies, underscoring the need for more targeted treatment strategies [3].

Catheter-based interventional therapies have gained recognition as potential reperfusion strategies in selected patients with IHR PE by enabling targeted thrombus reduction while limiting systemic exposure to thrombolytic agents [1,16,17]. Available evidence suggests short-term haemodynamic improvement following intervention; however, uncertainty remains regarding their impact on clinically meaningful outcomes and optimal patient selection [18].

Existing reviews have primarily focused on device characteristics and procedural outcomes associated with catheter-based therapies. In contrast, this narrative review aims not only to summarise available interventional strategies for IHR PE in adults, but also to provide a critical appraisal of the current evidence base and its methodological limitations. Particular emphasis is placed on the clinical heterogeneity of IHR PE, the limitations of static risk stratification, and the growing importance of patient selection in determining the role of catheter-based intervention. The review further explores a trajectory-based approach to treatment escalation, highlighting the discrepancy between haemodynamic surrogate outcomes and patient-centred clinical endpoints, as well as key evidence gaps that continue to limit routine implementation of catheter-based therapies. 

## 2. Methodology

This article was designed as a narrative review aimed at critically evaluating the current evidence regarding catheter-based therapies for IHR PE, with particular emphasis on catheter-directed thrombolysis (CDT), mechanical thrombectomy and patient selection strategies.

A literature search was conducted using PubMed, Scopus, Web of Science, and Google Scholar. Publications from 2013 to 2026 were considered. The search strategy utilised combinations of the following terms: “pulmonary embolism”, “intermediate-high-risk pulmonary embolism”, “submassive pulmonary embolism”, “catheter-directed therapy”, “catheter-directed thrombolysis”, “ultrasound-assisted thrombolysis”, “mechanical thrombectomy”, “aspiration thrombectomy”, “pulmonary embolism response team”, “right ventricular dysfunction”, and “reperfusion therapy”. Boolean operators (AND, OR) were applied as appropriate to optimise search sensitivity and specificity.

Studies were considered eligible if they evaluated catheter-based treatment strategies for acute PE in adults evaluated haemodynamic, procedural, or clinical outcomes associated with CDT or mechanical thrombectomy; addressed patient selection, procedural workflow, multidisciplinary management, or technological advancements in PE intervention; or provided clinically relevant mechanistic or guideline-based insights into IHR PE management. Priority was given to randomised controlled trials (RCTs), prospective studies, multicentre registries, guideline documents, and comprehensive review articles.

Exclusion criteria included studies focused exclusively on chronic thromboembolic pulmonary hypertension (CTEPH), paediatric populations, non-English-language publications, conference abstracts without full-text availability, and isolated technical reports with limited clinical relevance.

The identified literature was reviewed and synthesised qualitatively. As this manuscript was designed as a narrative review rather than a systematic review, formal PRISMA-guided study selection procedures, quantitative evidence synthesis, and structured risk-of-bias assessment tools were not applied. Nevertheless, particular attention was paid to study design, sample size, presence or absence of comparator groups, endpoint selection, duration of follow-up, consistency of findings across studies, and potential sources of bias. Special consideration was given to the predominance of registry-based data, industry-sponsored investigations, and the frequent reliance on surrogate haemodynamic endpoints, which may limit the interpretation and generalisability of reported outcomes.

The objective of this methodology was not to generate a fully reproducible systematic evidence synthesis but rather to provide a comprehensive and critical overview of the contemporary literature, identify important evidence gaps, and evaluate the clinical implications of currently available catheter-based treatment strategies in patients with IHR PE.

## 3. Haemodynamic and Pathophysiological Rationale of Interventional Therapies

Acute PE produces an abrupt increase in pulmonary vascular resistance (PVR) through both mechanical obstruction of the pulmonary vasculature and secondary vasoconstriction, resulting in elevated pulmonary artery pressure (PAP) and acute RV pressure overload [2,19,20]. Because the RV is poorly adapted to sudden increases in afterload, this may rapidly lead to RV dilation, dysfunction, and haemodynamic compromise [19,21].

In IHR PE, RV dysfunction is present alongside myocardial injury and indicates the early onset of haemodynamic instability [2,4,5], even in the absence of systemic hypotension [6,22].

This pathophysiological framework provides a plausible rationale for catheter-based reperfusion strategies aimed at rapidly reducing pulmonary vascular obstruction and RV afterload, thereby interrupting the cascade leading from RV overload to haemodynamic collapse [3,7]. This concept is particularly relevant because many IHR PE patients exist in a state of compensated or normotensive shock in which conventional haemodynamic parameters may underestimate underlying instability [6,11,12].

Moreover, the relationship between thrombus burden and clinical severity remains complex and incompletely understood [23]. It has been demonstrated that the extent of anatomical obstruction does not correlate linearly with haemodynamic compromise or mortality [24,25]. Clinical outcomes are strongly influenced by baseline RV reserve, cardiopulmonary comorbidities, and individual adaptive capacity [26]. Consequently, a reduction in clot burden alone may not necessarily translate into improved clinical outcomes [23], underscoring the fact that physiological improvement does not always equate to measurable patient benefit.

Although early reductions in right ventricular-to-left ventricular (RV/LV) ratio and PAP are consistently reported after catheter-based intervention, the extent to which these physiological improvements translate into meaningful long-term clinical benefit remains uncertain [17,27]. Therefore, while the pathophysiological rationale for early reperfusion is compelling, physiological plausibility alone should not be considered evidence of clinical superiority.

## 4. Limitations of Conventional Therapy

Conventional management of acute PE is centred around anticoagulation, which remains the first-line treatment in most patients [2,13]. Although anticoagulants effectively prevent thrombus propagation and recurrent embolic events [28], they do not directly remove existing thrombotic burden [29]. Instead, thrombus resolution depends on endogenous fibrinolysis, a process that may be slow and highly variable between patients [30].

This limitation partly explains the growing interest in catheter-based reperfusion strategies in IHR PE [27,31,32]. However, it remains uncertain whether delayed thrombus resolution alone justifies routine early intervention in haemodynamically stable patients [33]. A substantial proportion of patients with IHR PE improve with anticoagulation alone, and identifying the subgroup at highest risk of deterioration remains challenging [2,13,33].

Systemic thrombolysis represents a more aggressive reperfusion strategy aimed at accelerating thrombus dissolution [2]. By enhancing fibrinolysis, thrombolytic therapy may improve pulmonary perfusion, reduce PAP, and facilitate recovery of RV function [7,34]. However, the PEITHO trial demonstrated that although systemic thrombolysis reduced haemodynamic decompensation, this benefit was offset by increased risks of major bleeding and stroke, without clear mortality reduction [35]. These findings highlight the central therapeutic dilemma in IHR PE management.

Some investigators argue that mortality-focused analyses may underestimate clinically meaningful benefits such as prevention of haemodynamic collapse, whereas others emphasise that reduction in clinical deterioration may not justify severe bleeding complications in predominantly normotensive patients [7,36]. The central challenge is therefore not simply whether escalation is possible, but rather when the anticipated benefits of intervention outweigh treatment-related and procedural risks.

Current management strategies often delay escalation until overt clinical deterioration occurs [8]. However, waiting too long may reduce the opportunity for effective intervention, whereas premature escalation may expose patients who could stabilise with anticoagulation alone to unnecessary complications. The emergence of catheter-based therapies should therefore be viewed not merely as technological advancement, but as a response to a persistent clinical dilemma that conventional treatment strategies have not fully resolved. 

## 5. Catheter-Based Reperfusion Strategies in IHR PE: From Procedural Options to Personalised Intervention

Current clinical guidelines continue to recommend anticoagulation as the first-line treatment for most normotensive patients with IHR PE [2,13,37]. In this population, routine use of reperfusion therapy is generally not recommended [2,13]. Consequently, catheter-based interventional therapies are considered in selected patients demonstrating clinical deterioration, impending haemodynamic decompensation despite anticoagulation, contraindications to systemic thrombolysis, or other high-risk clinical features following multidisciplinary assessment [9,38].

Importantly, decision-making regarding escalation to catheter-based intervention remains highly individualised and is frequently guided by Pulmonary Embolism Response Teams (PERTs) [38,39]. Existing catheter-based reperfusion strategies include CDT, mechanical thrombectomy, and hybrid approaches [1,2,40,41]. However, despite increasing procedural use, the central challenge in contemporary interventional PE management is no longer the lack of available technologies but rather the identification of patients most likely to derive meaningful clinical benefit from intervention.

This highlights a fundamental limitation of current PE risk stratification frameworks. Although existing classifications effectively identify patients at increased risk of adverse outcomes, they provide limited guidance regarding the optimal timing and selection of specific interventional strategies. In clinical practice, treatment decisions rely on dynamic assessment of haemodynamic trajectory rather than static risk categories alone.

**Figure 1:**
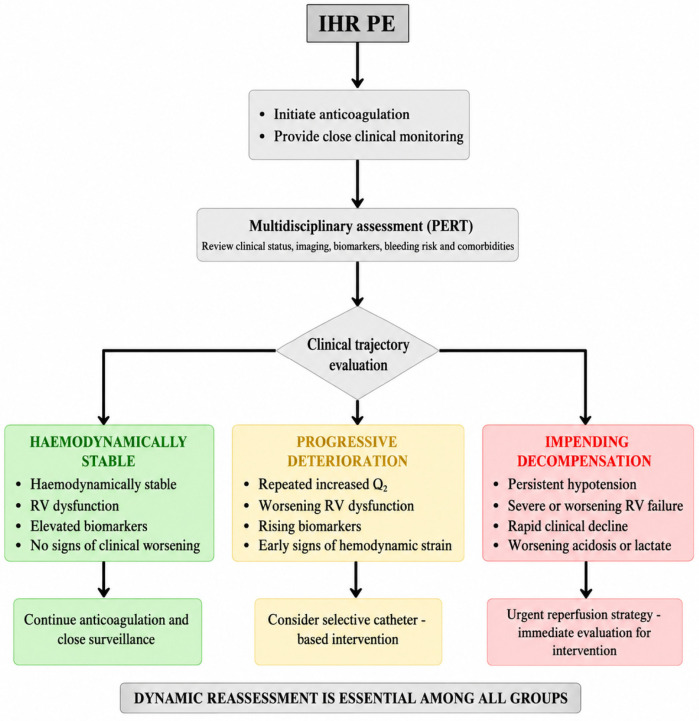
Proposed non-validated decision pathway for IHR PE, illustrating dynamic clinical trajectory assessment and potential management strategies. Key decision modifiers across all trajectories include bleeding risk, RV dysfunction, biomarker trends, clot burden, institutional expertise, and patient-specific factors such as comorbidities.

Clinically, IHR PE is better understood as a heterogeneous spectrum with variable trajectories of deterioration rather than as a single uniform category. Some patients remain stable despite RV dysfunction and myocardial injury, whereas others demonstrate gradual or rapid progression toward haemodynamic compromise. Pragmatically, this spectrum may be viewed as encompassing three broad trajectories: haemodynamically stable patients suitable for anticoagulation and surveillance, patients with progressive clinical worsening who may warrant consideration of catheter-based reperfusion, and patients with impending haemodynamic decompensation in whom urgent intervention becomes increasingly relevant. This heterogeneity reinforces the limitations of static risk stratification and supports a more individualised approach to treatment escalation. Figure 1 summarises a proposed non-validated trajectory-based clinical decision pathway for IHR PE, integrating haemodynamic progression, risk of deterioration, and potential escalation to catheter-based reperfusion strategies.

CDT is one of the catheter-based reperfusion strategies used in IHR PE. By enabling local delivery of low-dose fibrinolytic agents directly into the pulmonary arteries, CDT aims to reduce thrombotic burden while limiting systemic exposure to thrombolysis compared with full-dose systemic treatment [1,16,17,42,43]. This approach may provide a favourable balance between haemodynamic improvement and bleeding avoidance in selected patients who remain stable but demonstrate progressive RV dysfunction or early clinical decline [15,16,27].

Several prospective studies, including ULTIMA, SEATTLE II, and OPTALYSE PE, have demonstrated consistent improvements in surrogate haemodynamic markers following CDT, particularly reductions in RV/LV ratio and PAP [15,27,44,45]. However, interpretation of these findings requires caution. Most available studies were limited by small sample sizes, non-randomised or single-arm designs, and short follow-up periods [17,32,46,47,48]. Moreover, substantial heterogeneity exists across studies regarding thrombolytic dosing protocols, infusion duration, catheter type, and endpoint definitions, limiting direct cross-study comparisons [2,32].

Mechanical thrombectomy represents an alternative catheter-based reperfusion strategy used in selected centres [10,49]. Unlike CDT, thrombectomy enables direct clot extraction without fibrinolytic administration, making it more suitable for patients with elevated bleeding risk or contraindications to thrombolysis [28,37]. Its rapid onset of action may be relevant in patients with worsening RV dysfunction, escalating oxygen requirements, rising biomarkers, or early signs of haemodynamic deterioration despite anticoagulation [10,37,46,50]. However, thrombectomy is not a complication-free procedure as large-bore vascular access introduces risks of bleeding, vascular injury, haemolysis, arrhythmias, and distal embolisation [50,51,52,53,54].

Prospective studies and registries, including FLARE, FLASH, EXTRACT-PE, and STRIKE-PE, have consistently reported favourable short-term reductions in RV/LV ratio and pulmonary haemodynamic parameters following thrombectomy [3,10,44,55]. Nevertheless, important limitations remain. Most thrombectomy studies are single-arm investigations or registry-based analyses involving highly selected patient populations treated in experienced centres [2,32]. Many studies were industry-sponsored, raising concerns regarding sponsorship bias and selective outcome reporting [56]. In addition, comparator arms are frequently absent, limiting conclusions regarding comparative effectiveness against anticoagulation or CDT [2,32].

The PEERLESS trial provided comparative data between large-bore mechanical thrombectomy and CDT, reporting generally positive outcomes with thrombectomy [46]. However, interpretation requires caution, as the primary endpoint was largely driven by escalation of care and clinical deterioration rather than hard clinical endpoints such as mortality alone. At present, no interventional strategy has demonstrated definitive superiority in reducing hard clinical outcomes in IHR PE.

The major prospective trials and registries evaluating CDT and mechanical thrombectomy in IHR PE are summarised in Table 1 and Table 2, highlighting substantial heterogeneity in study design, endpoints, and methodological quality.

From a clinical perspective, CDT and mechanical thrombectomy should be viewed as complementary rather than competing strategies [62]. Their selection depends not only on bleeding risk and haemodynamic status, but also on thrombus burden, anatomical considerations, anticipated speed of deterioration, procedural feasibility, and institutional expertise (Table 3) [62,63]. In general, CDT may be more suitable in patients with preserved haemodynamic stability but progressive RV dysfunction, where controlled reperfusion is preferred [64,65]. In contrast, mechanical thrombectomy may be preferred in patients requiring rapid reperfusion, particularly those with impending decompensation, extensive central thrombotic burden, or contraindications to thrombolysis [22,63,66]. Importantly, the anatomical extent of embolic obstruction does not always correlate with haemodynamic severity, highlighting the limitations of anatomy-based procedural decision-making and reinforcing the importance of dynamic clinical assessment [24,25].

Hybrid approaches combining mechanical thrombectomy with low-dose CDT represent an emerging strategy intended to integrate the advantages of both techniques [38,42]. These approaches intend to achieve rapid thrombus reduction while minimising total thrombolytic exposure, potentially offering additional flexibility in patients with extensive thrombotic burden, incomplete response to single-modality treatment, or complex haemodynamic and anatomical presentations [18,59,74,75]. Although current evidence supporting hybrid approaches remains limited to observational studies and small clinical series, these techniques reflect a shift toward strategy-based rather than device-based interventions. 

Emerging technologies, including advanced imaging integration, haemodynamic monitoring, and artificial intelligence (AI)-assisted risk assessment, are being explored as potential tools to improve procedural planning and patient selection [73,76,77,78,79,80]. However, their clinical value remains largely investigational and requires prospective validation.

Despite substantial advances in catheter-based technologies, important uncertainties remain regarding patient selection, optimal timing of intervention, and integration of these therapies into contemporary treatment pathways. Future progress in IHR PE management will depend not only on generating higher-quality evidence but also on developing more refined approaches to risk stratification that identify patients most likely to benefit from early intervention.

## 6. Real-World Implementation and Practical Challenges

Successful implementation of catheter-based therapies in IHR PE depends not only on procedural efficacy, but also on timely diagnosis, institutional organisation, multidisciplinary coordination, and operator expertise [39,49,51]. In contemporary practice, efficient in-hospital pathways are increasingly viewed as relevant for minimising delays in treatment escalation, particularly in patients with evolving haemodynamic compromise [81].

Timely escalation remains one of the most challenging aspects of interventional PE management [8]. Delayed intervention may reduce the opportunity to prevent haemodynamic decompensation, whereas premature escalation may expose patients, who could stabilise with anticoagulation alone, to unnecessary procedural risks [8]. As a result, treatment decisions increasingly rely on multidisciplinary PERTs [32,38,39,82].

Substantial variability persists between centres in procedural volume, device availability, operator experience, and institutional protocols [57,83]. This variability has important implications for both procedural outcomes and external validity. Outcomes reported from high-volume centres with established PE programmes may not be fully generalisable to lower-volume institutions or resource-limited settings [32,84]. Consequently, differences in infrastructure and expertise remain important determinants of real-world treatment effectiveness.

Economic and logistical factors further influence implementation of catheter-based therapies in routine clinical practice [84]. Advanced thrombectomy platforms and specialised catheter systems are not universally available, and reimbursement structures vary considerably across healthcare systems [32,49,84]. Therefore, real-world treatment decisions are shaped not only by clinical indications, but also by institutional resources, procedural availability, and local expertise [49,83,85].

These implementation challenges highlight an important gap between procedural efficacy demonstrated in specialised centres and real-world applicability across broader healthcare settings. Accordingly, successful integration of catheter-based therapies into IHR PE management depends not only on technological advancement, but also on optimising patient selection, institutional readiness, and equitable access to specialised care.

## 7. Patient Selection: Toward Personalised Interventional Therapy

Determining which patients should undergo catheter-based reperfusion remains one of the most difficult aspects of contemporary IHR PE management [49]. Current guidelines recommend incorporating clinical scores, biomarkers, and imaging findings to identify patients at increased risk of adverse outcomes [2,9,38]. However, treatment decisions should extend beyond risk stratification alone and require integration of bleeding risk, procedural feasibility, comorbidities, and individual therapeutic goals.

Importantly, predictors of clinical deterioration do not necessarily correspond to predictors of procedural benefit [10,47,86]. Therefore, identifying patients at increased risk of adverse outcomes should be viewed as a necessary but insufficient step when considering escalation to catheter-based reperfusion.

In practice, four domains are particularly relevant when evaluating candidates for intervention: risk of deterioration, bleeding risk, procedural feasibility, and patient-centred goals of care [87]. Together, these factors often provide more clinically relevant guidance than risk classification alone.

Indicators of evolving clinical deterioration may include worsening RV dysfunction on serial imaging, progressive RV dilatation, increasing RV/LV ratio, rising cardiac biomarker concentrations, escalating oxygen requirements, elevated lactate levels, or early signs of haemodynamic instability despite preserved systemic blood pressure [87]. Although biomarkers such as troponin and NT-proBNP, together with echocardiographic and computed tomography findings, remain central components of risk assessment, no individual parameter provides sufficient specificity to guide intervention independently [2,38,88,89]. Accordingly, serial reassessment and integration of multiple clinical variables are often more informative than isolated measurements.

Bleeding risk represents another critical determinant of treatment selection. Patients with recent surgery, previous major haemorrhage, intracranial pathology, thrombocytopenia, or other contraindications to thrombolytic therapy may be less suitable for thrombolysis-based strategies and may require consideration of alternative approaches [2,10,49].

Procedural feasibility should also be considered, including vascular access, thrombus location, anatomical complexity, operator expertise, and institutional experience [62,63]. In practice, procedural success depends not only on patient characteristics but also on the capabilities of the treating centre.

Particular challenges arise in patient populations that remain underrepresented in contemporary interventional PE trials. Individuals with active malignancy, chronic kidney disease, pregnancy, advanced age, or frailty often present with competing thrombotic and bleeding risks that are not adequately reflected in existing evidence [90,91,92,93,94,95,96,97]. In these patients, therapeutic success may extend beyond conventional endpoints such as mortality or haemodynamic improvement and include preservation of functional status, avoidance of prolonged hospitalisation, and alignment with patient-centred goals of care.

Taken together, these considerations support a personalised approach to treatment selection in IHR PE. Rather than relying exclusively on baseline risk classification, contemporary decision-making increasingly requires integration of clinical evolution, procedural risk, patient characteristics, and individual treatment objectives within a multidisciplinary framework. 

## 8. Multidisciplinary Care

The growing complexity of therapeutic decision-making in IHR PE has led to increasing recognition of the importance of multidisciplinary care, particularly through PERTs [38,82]. These teams typically integrate expertise from acute care, cardiovascular medicine, imaging, and interventional specialties to support management of high-risk PE, IHR PE, and cases characterised by substantial therapeutic uncertainty [27,38,39,83].

The clinical value of PERTs lies not simply in coordinating treatment, but in addressing one of the central challenges in IHR PE management: balancing the risk of haemodynamic deterioration against bleeding risk, procedural feasibility, and overall patient-specific clinical context [83]. This is particularly relevant in IHR PE, where escalation decisions are often complex and time-sensitive, and where no single clinical variable reliably identifies patients most likely to benefit from intervention.

PERT involvement generally spans the entire clinical pathway, from early case identification and risk assessment to treatment selection, procedural planning, and post-intervention monitoring [83]. In addition, multidisciplinary teams may contribute to longitudinal follow-up, including evaluation for chronic thromboembolic complications such as CTEPH [98].

Available observational evidence suggests that PERT implementation may improve clinical decision-making and facilitate more efficient escalation of care. Meta-analytic data indicate associations between PERT involvement and shorter ICU stays, with lower mortality reported in some high-risk PE cohorts [82,98]. However, these findings should be interpreted cautiously, as current evidence is derived predominantly from observational studies and remains vulnerable to selection bias, institutional confounding, and centre-specific expertise effects.

Important practical limitations also remain. PERT implementation requires institutional resources, multidisciplinary availability, and efficient communication infrastructure, which may not be feasible in all healthcare settings. In addition, concerns have been raised regarding increased healthcare costs and potential over-reliance on specialised teams in settings where most patients remain clinically stable under standard management [98].

Despite these limitations, multidisciplinary care remains particularly valuable in IHR PE because it reflects the complexity and diversity of this patient population. As management increasingly shifts toward personalised and trajectory-based treatment strategies, multidisciplinary evaluation is likely to remain central to optimising patient selection, treatment timing, and procedural decision-making.

## 9. Discussion: Clinical Evidence and Future Directions in Interventional PE Management

### 9.1. Current Evidence, Methodological Limitations, and Unresolved Challenges

Despite increasing use of catheter-based therapies in IHR PE, the supporting evidence remains limited and methodologically heterogeneous, creating persistent uncertainty in clinical decision-making [63,99]. A defining feature of the current evidence base is the predominance of observational studies and prospective registries, which account for the majority of available data [17,100]. Although these studies frequently report improvements in haemodynamic parameters and markers of RV strain following CDT and mechanical thrombectomy [15,45], these findings primarily reflect short-term physiological effects rather than definitive clinical benefit [17].

Interpretation of the available evidence is further complicated by substantial heterogeneity in patient selection, procedural techniques, endpoint definitions, and follow-up duration across studies [2]. Recent meta-analyses and systematic reviews suggest that catheter-based interventions may be associated with haemodynamic improvement and reduced risk of clinical deterioration compared with anticoagulation alone [101,102], while mechanical thrombectomy may offer lower bleeding risk than systemic thrombolysis in selected populations [103,104]. However, these findings are derived predominantly from observational data and therefore inherit many of the limitations of the underlying evidence base. Consequently, current evidence remains insufficient to establish definitive comparative effectiveness or safety advantages between available treatment strategies (Table 4).

RCTs, which are essential for establishing therapeutic benefit, remain limited in number and are generally constrained by small sample sizes, short follow-up durations, and insufficient statistical power to assess mortality or other clinically meaningful outcomes [32,38,46,47,48].

Another major limitation is the continued reliance on surrogate endpoints [17]. Improvements in RV/LV ratio, PAP, and markers of RV strain are consistently reported and are frequently utilised as indicators of therapeutic success [27]. However, improvements in surrogate haemodynamic parameters should not be interpreted as evidence of improved patient-centred outcomes. To date, no adequately powered body of evidence has demonstrated that these physiological improvements translate into lower mortality, reduced recurrent PE, improved functional capacity, better quality of life, or reduced incidence of chronic thromboembolic complications.

Selection bias further limits interpretation of interventional studies. Patients enrolled in trials and registries are frequently treated in experienced, high-volume centres and often represent carefully selected populations. Individuals with extreme bleeding risk, advanced frailty, severe comorbidity burden, or complex clinical presentations are commonly underrepresented. As a result, reported procedural safety and efficacy may not accurately reflect outcomes achievable in broader real-world populations, particularly as catheter-based therapies are increasingly adopted outside specialised centres.

Despite these persistent limitations in the clinical evidence base, technological innovation continues to influence catheter-based management strategies for PE [32,63]. However, technological advancement alone should not be assumed to confer superior clinical outcomes. Many contemporary devices have entered clinical practice based on favourable haemodynamic outcomes and procedural feasibility rather than definitive evidence demonstrating improved survival, long-term functional recovery, or better patient-centred outcomes. In addition, substantial industry involvement in device development, registry maintenance, and clinical investigation introduces potential risks of sponsorship bias, selective outcome reporting, and preferential study design [56]. Although industry-supported studies have contributed important feasibility and safety data, their findings should be interpreted within the broader context of independent evidence whenever possible.

Overall, the interventional management of IHR PE remains a rapidly evolving field characterised by substantial technological progress but persistent evidentiary uncertainty. At present, the strongest evidence supports the ability of catheter-based therapies to improve short-term haemodynamic parameters in selected patients. Whether these physiological benefits justify broader routine intervention in IHR PE remains uncertain. Until adequately powered comparative trials demonstrate clear improvements in clinically meaningful outcomes, catheter-based therapies should be viewed as promising but incompletely validated treatment strategies.

### 9.2. Personalised Patient Selection and a Trajectory-Guided Approach to Intervention

Despite substantial technological progress, a persistent disconnect remains between favourable haemodynamic effects reported in interventional studies and the absence of convincing evidence for improved patient-centred outcomes. This gap may reflect not only limitations in the available evidence, but also suboptimal patient selection for intervention.

Most existing studies have been designed to evaluate procedural performance and short-term physiological outcomes. As a result, contemporary treatment algorithms remain largely device-centred and risk-category-driven [2,37]. However, these approaches provide limited insight into the fundamental clinical question: which patients should undergo intervention, and at what stage of disease evolution?

This distinction may help explain the persistent discrepancy between favourable haemodynamic outcomes and the absence of convincing evidence for improved patient-centred endpoints. It is plausible that the benefit of intervention depends not only on the type of therapy delivered, but also on the timing of intervention within an individual patient’s clinical trajectory.

From this perspective, IHR PE may be viewed not as a single therapeutic entity but as a dynamic clinical process. Some patients experience spontaneous stabilisation with anticoagulation alone, whereas others progress toward worsening RV dysfunction and eventual haemodynamic collapse despite initially similar presentations [2,11,13,33,87,105]. Consequently, the same intervention may confer substantially different benefit depending on the trajectory on which it is applied.

This concept suggests a potential shift from static risk-category-based treatment algorithms toward trajectory-guided intervention strategies. Rather than asking whether all patients classified as IHR should be considered for reperfusion, future studies may need to determine which evolving clinical phenotypes derive meaningful net benefit from escalation of therapy before irreversible RV failure develops.

Under such a framework, the primary objective of future research would move beyond comparative device evaluation. Instead, emphasis would be placed on identifying predictors of treatment responsiveness, validating trajectory-specific intervention thresholds, and developing models capable of integrating serial clinical, imaging, and biomarker data into personalised treatment decisions.

Importantly, this hypothesis remains unproven and should not be interpreted as a validated treatment algorithm. However, it provides a conceptual framework that may help address one of the major limitations of current evidence: the assumption that patients within the same risk category represent a relatively homogeneous therapeutic population.

Ultimately, the future of interventional PE management may depend less on developing increasingly sophisticated devices and more on understanding when intervention changes the natural history of disease. In this context, the right question may no longer be which device should be used, but rather which patient should be treated, and when.

## 10. Conclusions

Catheter-based reperfusion therapies have emerged as promising treatment strategies for selected patients with IHR PE, offering the potential for rapid haemodynamic improvement while potentially reducing the bleeding risks associated with systemic thrombolysis. Available evidence consistently demonstrates favourable effects on surrogate haemodynamic parameters, including RV function and PAP. However, robust evidence supporting improved mortality and other patient-centred clinical outcomes remains limited.

A major challenge in contemporary IHR PE management lies not only in determining which interventional strategy to use, but also in identifying which patients are most likely to benefit from treatment escalation and when intervention should occur. Current risk stratification models remain essential for prognostic assessment but provide limited guidance for individualised interventional decision-making in this clinically heterogeneous population.

Future progress in interventional PE management will likely depend on improved patient selection, more refined risk assessment, and better understanding of disease trajectory. A more personalised, trajectory-guided approach integrating clinical evolution, imaging findings, biomarker dynamics, and multidisciplinary assessment may help optimise treatment decisions and improve clinically meaningful outcomes. Well-designed prospective studies and adequately powered randomised trials remain essential to define the role of catheter-based therapies in IHR PE more precisely.

## Figures and Tables

**Table 1:** Major clinical trials and registries evaluating catheter-based interventional therapies for IHR PE: study designs, patient populations, interventions, and primary endpoints. MAEs—major adverse events (e.g., device-related death, major bleeding, device-related clinical deterioration, device-related pulmonary vascular injury, and device-related cardiac injury). USAT—ultrasound-assisted thrombolysis.

Study	Study Design (Sample Size)	PE Risk Category	Intervention/Device	Comparator	Follow-Up	Primary Endpoint	References
**ULTIMA**	RCT (59)	Intermediate-risk PE	USAT + anticoagulation	Anticoagulation alone	90 days	Change in RV/LV ratio at 24 h	[44]
**SEATTLE II**	Prospective single-arm study (150)	Massive and submassive PE	USAT	None	30 days	RV/LV ratio reduction at 48 h	[45]
**OPTALYSE PE**	Randomised dose-optimisation trial (101)	Intermediate-risk PE	USAT (various alteplase regimens)	No external comparator	1 year	RV/LV ratio reduction	[57]
**SUNSET sPE**	Randomised trial (82)	Submassive PE	Standard CDT vs. USAT	Active comparator	90 days	Thrombus reduction	[58]
**FLARE**	Prospective single-arm study (106)	Intermediate-risk PE	FlowTriever (Inari Medical, Irvine, CA, USA) mechanical thrombectomy	None	30 days	RV/LV ratio reduction at 48 h	[3]
**FLASH**	Prospective registry (>800)	Predominantly intermediate-risk PE	FlowTriever thrombectomy	None	6 months	Composite of MAEs within 48 h	[10]
**EXTRACT-PE**	Prospective single-arm study (119)	Submassive PE	Indigo aspiration system (Penumbra, Inc., Alameda, CA, USA)	None	30 days	RV/LV ratio reduction at 48 h	[55]
**STRIKE-PE**	Prospective registry (150+)	Predominantly intermediate-risk PE	Mechanical thrombectomy	None	90 days and 365 days	RV/LV ratio reduction and MAEs	[59]
**PEERLESS**	RCT (550)	Intermediate-risk PE	Large-bore mechanical thrombectomy	CDT	30 days	Composite hierarchical endpoint	[60]
**HI-PEITHO**	Ongoing RCT	IHR PE	CDT + anticoagulation	Anticoagulation alone	Ongoing	PE-related death, haemodynamic collapse, recurrent PE	[61]

**Table 2:** Clinical outcomes, safety profiles, and key limitations of major trials and registries evaluating catheter-based therapies for acute PE.

Study	RV/LV Ratio Change	Mortality	Major Bleeding/ICH	Recurrent PE/Clinical Deterioration	Main Limitations	References
**ULTIMA**	RV/LV ratio reduction (baseline → 24 h): 1.28 ± 0.19 → 0.99 ± 0.17 (USAT); 1.20 ± 0.14 → 1.17 ± 0.20 (heparin)	No PE- or procedure-related deaths within 30 days	No major bleeding or ICH	No recurrent PE difference	Small sample size; surrogate endpoint-focused	[44]
**SEATTLE II**	RV/LV ratio reduction (baseline–48 h): 1.55 → 1.13; mean difference −0.42; *p* < 0.0001	30-day mortality 2.7%	Bleeding (10%); no ICH	Clinical improvement observed	No comparator arm	[45]
**OPTALYSE PE**	RV/LV ratio (baseline → change at 48 h): Arm 1: 1.47 ± 0.39 → −0.40 ± 0.37; Arm 2: 1.43 ± 0.33 → −0.35 ± 0.27; Arm 3: 1.49 ± 0.37 → −0.42 ± 0.32; Arm 4: 1.51 ± 0.58 → −0.48 ± 0.51	30-day mortality 1%	Major bleeding: 4%; ICH: 2% (1% attributed to tPA delivered by USCDT)	Limited clinical events	Small sample size; surrogate outcomes	[57]
**SUNSET sPE**	RV/LV ratio (baseline → reduction at 48 h): USAT: 1.54 ± 0.30 → 0.37 ± 0.34; CDT: 1.69 ± 0.44 → 0.59 ± 0.42	hospital mortality −2.5%	Major bleeding: 5%; ICH: none	Similar clinical outcomes	Underpowered for clinical endpoints	[58]
**FLARE**	RV/LV ratio reduction at 48 h: 0.38 (25.1%)	30-day mortality: 1% (due to undiagnosed breast cancer)	Major bleeding—1%	Clinical improvement observed	Single-arm design; short follow-up	[3]
**FLASH**	48 h: RV/LV ratio decreased from 1.23 ± 0.36 to 0.98 ± 0.31	48 h all-cause mortality: 0.3%; 30-day all-cause mortality: 0.8%; no device-related deaths	Bleeding rates—1.4%	Clinical deterioration—0.3%	Registry design; no randomisation	[10]
**EXTRACT-PE**	48 h: mean RV/LV ratio reduction 0.43 from baseline 1.47 ± 0.30	30-day all-cause mortality: 2.5%; device-related death: 1.7%	Bleeding rates—1.7%	Clinical deterioration 1.7%	No comparator group	[55]
**STRIKE-PE**	48 h: RV/LV ratio decreased from 1.39 to 1.01 (25.7% reduction)	30-day all-cause mortality: 2.0%	Major bleeding—2.7%	Device-related clinical deterioration—1.3%	Registry-based data	[59]
**PEERLESS**	RV/LV reduction (LBMT vs. CDT) 0.32 ± 0.24 vs. 0.30 ± 0.26)	30-day mortality (LBMT vs. CDT): 0.4% vs. 0.8%.	Bleeding rates (LBMT vs. CDT): major bleeding 6.9% vs. 6.9%, ICH 0.7% vs. 0.4%	Lower escalation of care with thrombectomy	Hierarchical composite endpoint-driven outcomes	[60]
**HI-PEITHO**	Pending	Pending	Pending	Pending	Results not yet available	[61]

**Table 3:** Comparison of CDT and mechanical thrombectomy in PE. Presented clinical scenarios are intended as general considerations rather than definitive indications. In contemporary practice, the choice between CDT and mechanical thrombectomy is highly individualised and depends on multiple patient-, procedure-, and institution-related factors. ICU—intensive care unit.

Feature	CDT	Mechanical Thrombectomy	References
**Mechanism**	Local low-dose fibrinolysis	Physical clot removal	[16,42]
**Use of thrombolytics**	Yes (alteplase usually 8–24 mg)	No	[9,67]
**Time to effect**	Gradual (hours) (6–24 h infusion)	Immediate (effect during procedure)	[3,10,51,52,68]
**Hemodynamic improvement**	Moderate–rapid	Rapid	[16,58,67]
**Bleeding risk**	Lower than systemic lysis (1–9.2% major bleeding depends on used catheter to 10% in systemic thrombolysis)	No thrombolytic -related bleeding, but access- and device-related bleeding remains possible (1–3% depending on the study)	[42,63,69,70,71]
**Hospitalisation in ICU**	98.6%	41.6%	[60]
**RV/LV reduction rate**	0.3+/−0.26 (without significant differences)	0.32+/−0.24	[42,60]
**Clinical scenarios where the approach may be considered**	Haemodynamically stable patients with RV dysfunction, acceptable bleeding risk, and no need for immediate reperfusion	Patients requiring rapid reperfusion, those with contraindications to thrombolysis, or selected cases with worsening clinical status after multidisciplinary evaluation	[42,72]
**Procedural complexity**	Moderate	Moderate-high	[43,67]
**Device dependency**	Moderate	High	[42,63]
**Limitations**	Still thrombolysis-related risks	Large-bore access, procedural risks	[43,56,73]

**Table 4:** Current evidence, methodological limitations, and remaining knowledge gaps in catheter-based treatment of IHR PE.

Clinical Domain	Current Evidence	Main Limitations	Research Priority	References
RV/LV ratio improvement	Consistently demonstrated	Surrogate endpoint	Link to clinical outcomes	[27]
Haemodynamic improvement	Frequently observed	Mostly single-arm studies	Comparative RCTs	[18,101,102]
Clinical deterioration	Emerging evidence	Limited RCT data	Validation in larger trials	[101,102]
Mortality reduction	Uncertain	Underpowered studies	Adequately powered RCTs	[17,21,35]
CTEPH prevention	Unknown	Lack of long-term follow-up	Longitudinal studies	[17,27]
Quality of life	Very limited evidence	Rarely assessed	Patient-centred outcomes	[17,59]
Patient selection	Poorly defined	Heterogeneous populations	Phenotype- and trajectory-based studies	[18,49]

## Data Availability

No new data was created or analysed in this study. Data sharing is not applicable to this article.

## Abbreviations

The following abbreviations are used in this manuscript:

PE	pulmonary embolism
IHR	intermediate-high-risk
RV	right ventricular
RV/LV ratio	right ventricular-to-left ventricular ratio
PAP	pulmonary artery pressure
CTEPH	chronic thromboembolic pulmonary hypertension
CDT	catheter-directed thrombolysis
USAT	ultrasound-assisted thrombolysis
ICU	intensive care unit
RCT(s)	randomised controlled trial(s)
PERT(s)	pulmonary embolism response team(s)
ICH	intracranial haemorrhage
MAEs	major adverse events
